# Focused Analysis of Complications Associated with Bovine Xenohybrid Bone Grafts Following Maxillary Sinus Augmentation via the Lateral Approach: A Retrospective Cohort Study

**DOI:** 10.3390/diagnostics15162089

**Published:** 2025-08-20

**Authors:** Pascal Grün, Marius Meier, Alexander Anderl, Christoph Kleber, Flora Turhani, Tim Schiepek, S. M. Ragib Shahriar Islam, Sebastian Fitzek, Patrick Bandura, Dritan Turhani

**Affiliations:** 1 Center for Oral and Maxillofacial Surgery, Department of Dentistry, Faculty of Medicine and Dentistry, Danube Private University, Steiner Landstrasse 124, 3500 Krems an der Donau, Austria; pascal.gruen@dp-uni.ac.at (P.G.); meier_marius_1@hotmail.com (M.M.); anderlalex123@gmail.com (A.A.); turhani.flora@dp-uni.eu (F.T.); schiepek.tim@dp-uni.eu (T.S.); patrick.bandura@dp-uni.ac.at (P.B.); 2 Clinical Application of Artificial Intelligence in Dentistry (CAAID) Group, Department of Dentistry, Faculty of Medicine and Dentistry, Danube Private University, Steiner Landstrasse 124, 3500 Krems an der Donau, Austria; 3 Dental Practice, 9490 Vaduz, Liechtenstein; 4 Division for Chemistry and Physics of Materials, Department of Medicine, Faculty of Medicine and Dentistry, Danube Private University, Steiner Landstrasse 124, 3500 Krems an der Donau, Austria; christoph.kleber@dp-uni.ac.at; 5 Austrian Center for Medical Innovation and Technology, 2700 Wiener Neustadt, Austria; ragib.shahriar@acmit.at; 6 Research Center for Clinical AI-Research in Omics and Medical Data Science (CAROM), Department of Medicine, Danube Private University (DPU), 3500 Krems an der Donau, Austria; 7 Center for Medical Physics and Biomedical Engineering, Medical University Vienna, 1090 Vienna, Austria; 8 Health Services Research Group, Medical Images Analysis and Artificial Intelligence (MIAAI), Department of Medicine, Faculty of Medicine and Dentistry, Danube Private University, Steiner Landstrasse 124, 3500 Krems an der Donau, Austria; sebastian.fitzek@dp-uni.ac.at

**Keywords:** maxillary sinus floor augmentation (MSFA), bovine xenohybrid grafts, dental implant, CBCT, graft-related complication

## Abstract

**Background**: Maxillary sinus floor augmentation (MSFA) is commonly used to increase posterior maxillary bone volume prior to implant placement. Although generally successful, late complications can impact long-term outcomes. The purpose of the study was to estimate the incidence and timing of atypical late complications following (MSFA) using bovine xenohybrid bone grafts. The study also aimed to evaluate whether preoperative bone volume is associated with the risk of complications. **Methods**: This retrospective cohort study was conducted at the Center of Oral and Maxillofacial Surgery, Danube Private University, Krems-Stein, Austria, and included patients who underwent MSFA with bovine xenohybrid bone grafts and either simultaneous or staged implant placement between January 2020 and December 2023. Preoperative bone volume of the posterior maxilla measured via cone beam computed tomography (CBCT) in the planned implant insertion position. The primary endpoint was the time (days) from MSFA to the occurrence of a graft-related complication (defined as atypical if occurring more than 6 months after MSFA and not related to peri-implantitis) The covariates included subjects’ age, sex, the quantity of graft used for MSFA, timing of dental implant insertion (simultaneous vs. staged) and implant dimensions. Kaplan–Meier analysis and Cox proportional hazards regression were used to evaluate time-to-event data. Only one graft site per patient was analyzed. **Results**: Atypical complications occurred in 9 out of 47 patients (19.1%), with an average time to onset of 645 days. In a multivariable analysis, a lower preoperative bone volume was found to be an independent predictor of an increased risk of complications (hazard ratio [HR]: 0.972; 95% confidence interval [CI]: 0.925–1.021; *p* = 0.252). However, the quantity of graft used for MSFA was not found to be a predictor (*p* = 0.46). **Conclusions**: Within the limitations of a retrospective study, reduced native bone volume appears to increase the risk of atypical late complications following MSFA with bovine xenohybrid grafts. This makes closer clinical and radiologic follow-up of patients over a longer period very necessary.

## 1. Introduction

Maxillary sinus floor augmentation (MSFA), first described by Tatum in 1977 [1] and later published by Boyne and James in 1980 [2], is a highly reliable and effective technique for compensating bone height loss in the posterior maxilla, enabling implant placement. The documented implant success rate with MFSA is 97.2% [3,4].

Implants can be placed either simultaneously with MSFA (one-stage) or after healing (staged), depending on the available bone height [5,6]. Over the past decade, various grafting materials have been evaluated for MSFA [7]. Autogenous bone is considered the gold standard due to its osteogenic potential [8]. However, xenogeneic bone grafts, particularly bovine-derived materials, are widely used due to their availability, biocompatibility, and volume stability [9].

Recently, xenohybrid bone grafts, such as SmartBone^®^ (Industrie Biomediche Insubri SA, Mezzovicco, Switzerland), have been developed for use in oral surgery. SmartBone^®^ combines a bovine mineral matrix with synthetic poly (L-lactide-co-ε-caprolactone) and RGD-exposed collagen fragments from porcine gelatin [10,11,12,13]. These modifications enhance elasticity, cell attachment, and integration while maintaining a porous structure conducive to vascular ingrowth [14].

Although preclinical and early clinical studies have demonstrated favorable osteoconductive properties of SmartBone^®^, recent clinical reports have described rare, material-related late complications, such as graft instability or chronic inflammation, raising concerns regarding its long-term safety [15,16,17].

MSFA may lead to intraoperative complications, such as perforation of the Schneiderian membrane (7–44%) [18], and postoperative complications, which are categorized as being early (within 3 weeks) or late (months to years) [19]. Clinical manifestations include swelling, fistulas, pain, suppuration, or even graft failure. The late complications are typically associated with peri-implantitis but may also occur independently due to material failure or graft infection [20,21,22].

In a recent study, atypical late complications that occurred independently of peri-implantitis were described, suggesting a possible association with graft material properties [20]. Understanding the risk factors for these complications is critical, particularly in patients with low preoperative bone volume, where larger graft volumes are typically required [20]. However, the role of bone volume and its relationship to complications remains unclear.

Complications occurring after MSFA can be categorized into early (occurring during the procedure or during the initial healing period) and late (occurring after 3 weeks following the procedure, with most of them presenting with peri-implantitis) complications. Sinus graft complications that occur months after MSFA, independent of peri-implantitis, with unclear sources of infection, or presenting with atypical clinical features are referred to as atypical late complications in the latest literature.

The objective of this retrospective cohort study was to investigate whether reduced native bone volume is associated with an increased risk of atypical late complications following maxillary sinus floor augmentation with bovine xenohybrid grafts. We hypothesized that patients with lower preoperative bone volume would experience a higher incidence of late complications compared to those with more residual bone.

Specifically, our study aimed to (1) estimate the time-to-event incidence of atypical complications following MSFA; (2) evaluate the association between preoperative bone volume and complication risk; (3) assess whether graft quantity used for MSFA is independently associated with complication occurrence; and (4) compare complication rates between simultaneous and staged implant placement protocols.

## 2. Materials and Methods

### 2.1. Study Design

This retrospective cohort study was approved by the central Ethical Review Board of the federal state of Lower Austria (approval number: GZ: DPU-EK/069) and conducted in accordance with the Declaration of Helsinki (2008 revision). All patients provided written informed consent before participating in the study. Patient records from 1 September 2020 to 30 June 2023 were reviewed at the Department of Oral and Maxillofacial Surgery, Danube Private University, Krems-Stein, Austria.

Inclusion criteria were age ≥ 18 years, lateral-approach MSFA using bovine xenohybrid bone grafts (SmartBone^®^), availability of standardized CBCT images prior to MSFA and complete basic information, medical records, and follow-up data. Exclusion criteria included a history of sinus floor elevation before surgery, sinus pathology (e.g., cyst or tumor), history of allergies (pollen, dander, mold and/or dust), systemic bone metabolism disorders (e.g., uncontrolled diabetes mellitus or osteoporosis), heavy smoking habit (>10 cigarettes/day), or incomplete follow-up data or imaging or CBCT scans exhibited artifacts or lack of clarity that could affect accurate measurements.

### 2.2. Surgical Procedures and Follow-Up Examinations

**Figure 1:**
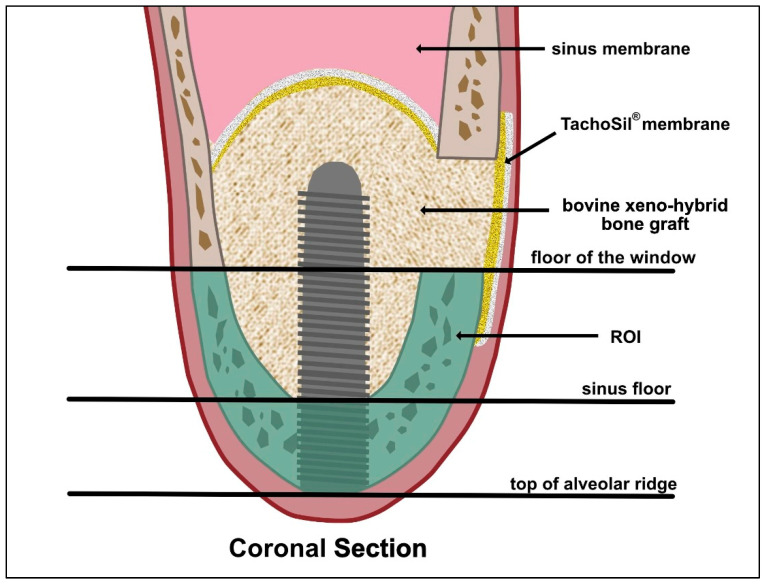
Schematic representation of the MSFA and the definition of the region of interest (ROI).

All patients underwent MSFA surgery performed by the same experienced surgeon from the Centre for Oral and Maxillofacial Surgery, as detailed in an earlier publication by our study group (Figure 1) [23]. Shortly, under local anesthesia (articaine 4% with adrenalin 1:100,000) with strict sterile conditions, incisions along the middle of the alveolar ridge and the buccal mesial and distal sides of the edentulous area were first made to elevate the full-thickness mucoperiosteum flap. Then the mucoperiosteal flap was elevated 12–15 mm above the alveolar ridge to expose the lateral wall of the maxillary sinus. Subsequently, a bone window was created by removing the lateral wall of the maxillary sinus using a circular diamond ball drill. The position of the lateral window was about 3 mm above the sinus floor, and the height of the window was about 6–8 mm. The maxillary sinus membrane was carefully peeled off and elevated using the sinus membrane elevators carefully and gently through the lateral approach under direct vision. The integrity of the Schneider membrane was checked by the Valsalva maneuver. Then, the maxillary sinus cavity was filled with 0.5–2.0 g bovine xenohybrid bone grafts (SmartBone^®^). After dental implant placement, a resorbable collagen membrane (TachoSil^®^, Corza Medical, Roggwil, Swiss, Switzerland) was covered on the outside of the window. Finally, the flap was precisely sutured (5.0 non-resorbable polypropylene suture) with utmost care to ensure secure closure and promote optimal healing conditions. All patients received oral health education and were instructed to use antibiotics amoxicillin/clavulanate acid 875 mg/125 mg (Augmentin^®^) (GlaxoSmithKline, London, UK), 2 times a day for 5 days or for penicillin allergy clindamycin (Dalacin C^TM^ 300 mg) (Pfizer, New York, NY, USA) 3 times a day for 5 days, 0.12% chlorhexidine gargle, compound furacilin nasal drops and acetaminophen tablets for 5–7 days. Ten days postoperatively the sutures were removed. After the operation, a CBCT scan was performed to assess the three-dimensional position of the implant and the condition of the graft material.

The follow-up examinations consisted of regular clinical and radiological assessments every six months, conducted by a university professor specializing in oral and maxillofacial surgery. The clinical follow-up examination of dental implants included an evaluation of the peri-implant soft tissue for signs of inflammation, such as erythema, swelling, or bleeding on probing. Additionally, probing depth measurements were performed, and radiological monitoring of the peri-implant bone level was conducted. Radiological follow-up was performed exclusively using annual CBCT scans. This high-resolution imaging technique allows for precise evaluation of peri-implant bone stability, detection of marginal bone loss, and assessment of the maxillary sinus graft. Radiological findings such as radiolucent or heterogeneous areas within the graft may indicate sinus graft infections, graft voids, or surgical ciliated cysts [24].

### 2.3. Radiomics Analysis: Image Segmentation and Volume Measurement

**Figure 2:**
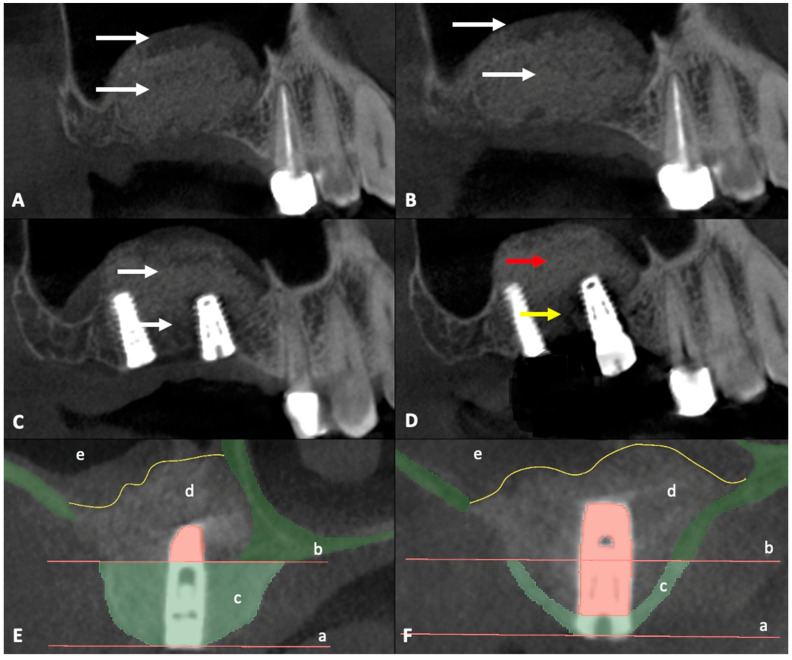
CBCT data obtained before and after surgery and ROI measurements with 3D Slicer image computing platform. (**A**) Postoperative CBCT image obtained after MSFA. No abnormalities were observed during the healing process. White arrow: Region of integrated graft material and an inflammation-free maxillary sinus. (**B**) Preoperative CBCT image obtained before the insertion of dental implants. No signs of pathology were present. (**C**) Postoperative CBCT image obtained after the insertion of dental implants. (**D**) Postoperative CBCT image obtained 8 months before implant placement. Red arrow: Homogeneous graft material, without evidence of infection or fistula formation. Yellow arrow: Region of non-integrated graft material lacking osseointegration. (**E**) ROI with sufficient bone availability. a: Alveolar crest; b: Inferior border of the lateral window; c: ROI; d: Xenohybrid bone graft; e: Sinus membrane; yellow line: Tachosil^®^ membrane; green part: autogenous bone. (**F**) ROI with limited bone availability. Labeling as in (**E**).

CBCT data obtained before surgery were combined with CBCT data obtained after surgery and dental implant position planning data to define the region of interest (ROI) (Figure 1). CBCT data were exported from the Sidexis 4 program (Dentsply Sirona, Bensheim, Germany) and imported into the 3D Slicer image computing platform (v.5.6.2, https://slicer.org, accessed on 22 October 2024). The measurements were performed by two independent experienced oral and maxillofacial surgeons. The CBCT images were aligned so that the implant was centered in the cross-section view. The ROI was delineated by drawing a line at the lower edge of the bone window, ensuring a 90-degree angle. The second line was drawn parallel to the first line at the crestal edge of the alveolar bone (Figure 1 and Figure 2E,F). Segmentation accuracy was verified, and manual adjustments were made if necessary. The existing bone was then segmented within the boundary lines using the Draw and Erase Tool and quantified using the Segment Statistics Tool.

### 2.4. Predictor Variable

The main predictor was the preoperative bone volume (in mm^3^) of the posterior maxillary alveolus. This was measured using CBCT scans obtained prior to MSFA in the planned implant insertion position (ROI). Volume segmentation was performed using 3D Slicer image computing platform by two independent reviewers (Figure 2E,F).

### 2.5. Outcome Variable

The primary outcome was the time (in days) from MSFA to the first occurrence of an atypical complication defined for the study.

For the purposes of this study, an atypical complication was defined as a clinically and/or radiographically confirmed graft-related adverse event that required surgery or was associated with persistent symptoms and that occurred more than 6 months after MSFA. This is not related to peri-implantitis and has unusual clinical or radiographic features (e.g., persistent graft instability, unexplained sinus opacity, chronic inflammation without infection, or fistula formation)

Typical late complications, such as peri-implantitis and early complications within the first 6 months were excluded from the analysis in this study.

### 2.6. Covariates

Covariates included age (years), sex (male/female), implant dimensions: diameter (mm) and length (mm), the quantity of graft used for MSFA (mm^3^), timing of dental implant insertion (simultaneous with MSFA vs. staged), MSFA laterality (unilateral (left/right) or bilateral), sudden onset of pain (yes/no, reported during follow-up examinations), and radiologic findings (yes/no based on CBCT imaging). The anatomical location of the implant (e.g., first molar, second molar) was recorded for each case; however, due to the limited sample size, no separate statistical analysis by site was performed.

### 2.7. Site Selection

To avoid statistical dependency, only one grafted site per patient was included in the final analysis. In cases of bilateral MSFA, the side with more complete follow-up data or a more severe clinical course was selected using predefined chart review criteria. Therefore, all statistical models were based on 47 unique graft sites (*n* = 47 patients).

### 2.8. Data Collection Methods

Electronic health records provided demographics, surgical details, and prosthetic timelines, which were de-identified and stored in a secure database. Prior to and after MSFA, CBCT digital imaging and communications in medicine files were imported into 3D Slicer version 4.x for volumetric segmentation by two independent reviewers, and all measurements were cross-validated.

### 2.9. Data Analyses

Continuous variables are presented as mean ± standard deviation (SD) or median with interquartile range (IQR) and categorical variables as counts and percentages. Kaplan–Meier analysis was used to estimate complication-free survival rate. Cox proportional hazards regression was applied to evaluate the associations between graft volume and complication timing, adjusting for covariates and accounting for bilateral clustering with robust sandwich estimators. Proportional hazards assumptions were checked using Schoenfeld residuals. Analyses were conducted in Python v3.12.6 using two-sided *p* < 0.05.

## 3. Results

### 3.1. Sample Characteristics

Forty-seven patients (26 female [55%], 21 males [45%]; mean age, 59.2 ± 8.3 years) met the inclusion criteria. Thirty-nine patients (83%) underwent unilateral grafting and eight (17%) underwent bilateral procedures. The median follow-up period was 754 days (IQR, 685–812 days).

A total of 84 implants were inserted (one implant/12 patients, two implants/33 patients, three implants/2 patients and only 1 patient did not receive a dental implant) from different manufacturers: Biomet 3i (ZimVie, Westminster, CO, USA): 30 T3^®^, 2 TPT^®^, 2 T3ST^®^; Neoss (Neoss, Cologne, Germany): 2 ProActive Tapered; BEGO (BEGO, Bremen, Germany): 36 S-Line, 2 Semados SC/SCX; SIC (SIC, Basel, Switzerland): 2 SiCmax Screw Implant; and Straumann (Institut Straumann AG, Basel, Switzerland): 8 BLX SL-Active. Regarding MSFA laterality, 26 MSFA were performed in the first quadrant and 21 in the second quadrant.

Regarding dental implant dimensions, implant lengths varied from 8.5 to 12 mm (1/8.5 mm, 2/9.5 mm, 70/10 mm, 7/11.5 mm and 3/12 mm). Similarly, implant diameters differed from 3.25 to 4.1 mm (5/3.25 mm, 4/3.5 mm, 39/3.75 mm, 31/4 mm and 4/4.1 mm).

Regarding quantity of bone substitute material used, 9 MSFA/0.5 g of the graft material was used, respectively, 2/0.75 g, 24/1 g, 6/1.5 g and 6/2 g.

### 3.2. Complication Incidence, Survival, and ROI Analysis

**Figure 3:**
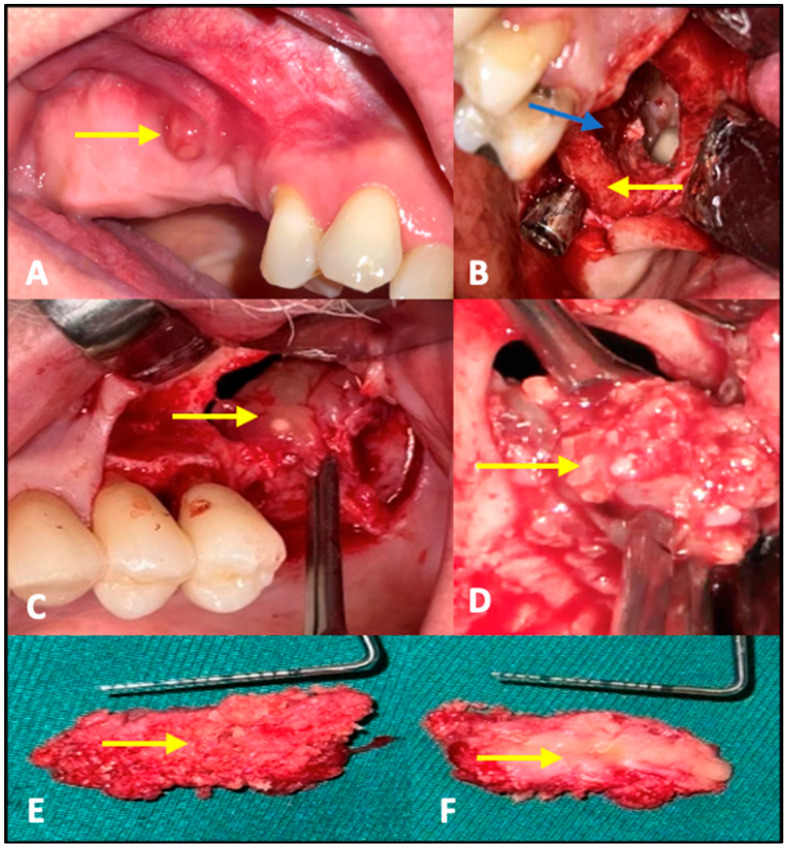
Clinical images documenting the complications. (**A**) Fistula formation 634 days after MSFA (yellow arrows); (**B**) Partial new bone formation around the implant apex (yellow arrows) and missing bone graft material with pus formation at the sinus floor (blue arrows) 842 days after MSFA. (**C**) Lack of new bone formation and swollen sinus membrane (yellow arrows) 781 days after MSFA. (**D**) Lack of new bone formation and removal of a mixture of bone graft particles and connective tissue from the sinus floor 940 days after MSFA. (**E**) Partly well-organized bone inlay from the cranial part of the bone substitute used 940 days before when performing MSFA in close contact with the sinus/Tachosil^®^ membrane (yellow arrows). (**F**) The back of this newly formed bone island shows scattered loose and unconsolidated bone substitute particles that are not osseointegrated (yellow arrows).

**Figure 4:**
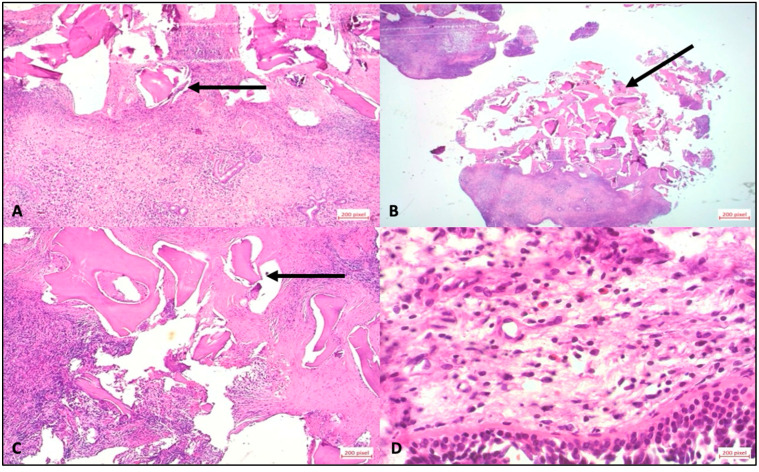
Histological images of a stained with hematoxylin and eosin using a staining device. (**A**) Bone fragments of different sizes, surrounded by connective tissue. Numerous lymphocytes and plasma cells in the parts of the stroma close to the mucosa; these inflammatory changes extend to the bone fragments close to the surface. (**B**) Bone fragments of different sizes; highly dense inflammatory stroma and epithelium. (**C**) Black arrow: Stroma with inflammatory cells, extending to the bone parts close to the mucosa. (**D**) Mucosal epithelium; minimally edematous stroma with small vessels; and lymphocytes, plasma cells and eosinophilic granulocytes in the stroma.

A total of 9 out of 47 patients (19.1%) developed atypical late complications and radiological evaluation of the ROI was performed to investigate the relationship between bone availability and complication risk. The median time to the first complication was 645 days (IQR 449–686). Atypical complications were distributed as follows in patients with MSFA and simultaneous implantation: two patients experienced pain between 322 (ROI 55.06 mm^2^) and 636 days (ROI 25.2 mm^2^) after surgery, thus delaying the prosthetic restoration. One patient suffered a loss of one of the two implants placed during the impression (distal implant with little residual bone (ROI 55.32 mm^2^ vs. ROI 13.12 mm^2^) 842 days after surgery (Figure 3B). In patients with MSFA and delayed implantation, one patient lost the implant and bone graft substitute at 752 days (ROI of 8.1 mm^2^). A second patient showed clinical discomfort at 640 days (ROI of 5.16 mm^2^). A third patient lost the implant during prosthetic impression at 612 days (ROI 14.07 mm^2^). A fourth patient exhibited a lack of osseointegration of the implant 938 days after MSFA (ROI 19.55 mm^2^). A fifth patient lost the implant and bone graft substitute at 781 days (ROI of 9.46 mm^2^) (Figure 3C). A sixth patient showed a fistula formation 634 days after surgery (Figure 3A) and exhibited a lack of osseointegration of the bone graft substitute; finally, the bone graft substitute was explanted after 940 days of MSFA (ROI of 0, considered non-measurable) (Figure 3D–F). In all the patients described, a decision was made to perform a new operation and remove bone replacement material due to the complications that occurred. Immediately after the surgery, the tissue was placed in sample containers containing 4.5% formaldehyde. Each sample was assigned a unique patient-specific identification number and macroscopically assessed according to size, color, consistency, and recognizable abnormalities. The tissue was then cut according to size and placed in an embedding cassette labeled with the patient number. According to standardized methods of sample preparation, the pathologist provided a diagnosis after examining the tissue using light microscopy and the polarization technique, which is used to detect foreign material with double refraction (Figure 4).

### 3.3. Kaplan–Meier Survival Analysis

**Figure 5:**
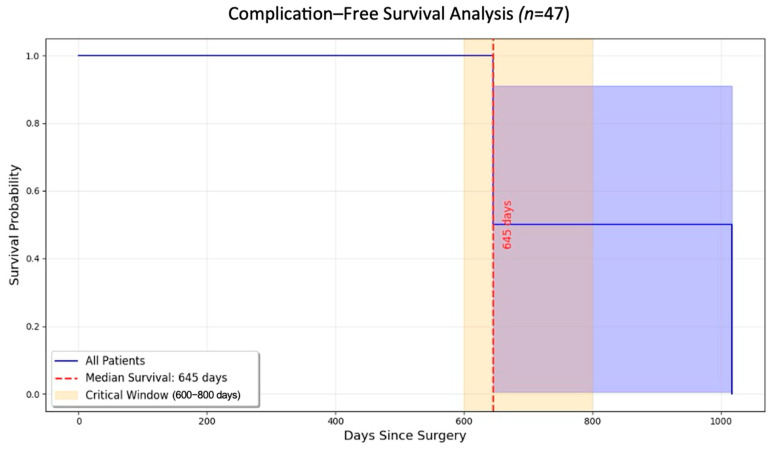
Kaplan–Meier survival analysis. This analysis showed a significant decrease in the probability of remaining complication-free over time. (The solid blue line = the estimated survival probability of being complication-free over time; The shaded blue area = the statistical uncertainty (confidence interval) around that estimate).

The Kaplan–Meier survival analysis revealed a significant decrease in the likelihood of remaining complication-free over time. Most complications were observed between 600 and 800 days after the procedure. The survival curve (Figure 5) captures the timeline for these complications and highlights the importance of extended follow-up care for patients undergoing MSFA. The median time to complications was 645 days, and a marked decline was observed in survival probability occurring within the critical 600–800-day period (log-rank *p* < 0.05). Beyond 800 days, patients who had not experienced complications exhibited stable outcomes and long-term success.

### 3.4. Quantity of Graft Used for MSFA Descriptive Statistics

The mean graft quantity was 1826 ± 742 mm^3^. Patients with complications had significantly larger graft quantity than those without complications (2250 ± 804 mm^3^ vs. 1680 ± 680 mm^3^; *p* = 0.002).

### 3.5. Risk Factor Analysis

In unadjusted analysis, each 100 mm^3^ increase in graft quantity was associated with a higher hazard of complication (hazard ratio [HR], 1.12; 95% confidence interval [CI], 1.07–1.18; *p* < 0.001). Detailed results of the univariate and bivariate analyses are presented in Table 1, Table 2 and Table 3.

A multivariable Cox proportional hazards regression was performed to evaluate the independent effects of preoperative bone volume (ROI), graft quantity, and other covariates on the risk of developing atypical late complications.

After adjusting for relevant clinical and procedural variables (including implant timing, age, and sex), lower preoperative bone volume (ROI) remained as a potential factor associated with a higher risk of atypical complications (HR 0.972 per 100 mm^3^ decrease; 95% CI: 0.925–1.021; *p* = 0.252).

In contrast, graft quantity did not retain statistical significance in the multivariable model (*p* = 0.46), indicating that its effect may be confounded by baseline bone availability. Although the hazard ratios for age and gender were not significant, a clear pattern emerged for the preoperative bone volume: a larger residual bone volume is associated with a lower risk of subsequent complications or implant loss. Specifically, the HR value per 30-day time unit is 0.972 (95% CI 0.925–1.021; *p* = 0.252). This non-significant trend nevertheless clearly emphasizes the decisive role of the available bone supply for the long-term success of treatment.

For a supplementary sensitivity test, we also tested a flat-rate 12/36-month approach. Here, too, the same trend was confirmed (HR 0.979; 95% CI 0.934–1.025; *p* = 0.357). The full results of the multivariable regression are summarized in Table 4.

## 4. Discussion

This study focused exclusively on late-onset atypical complications that were not directly related to peri-implantitis and occurred more than 6 months after performing MSFA with bovine xenohybrid bone grafts. Early complications and typical peri-implant failures were not included in the study. Our working hypothesis was that MSFA success is independent of bone volume and of the amount of augmentation material used.

We observed that graft material quantity did not correlate linearly with complication rates based on Spearman’s rank correlation; however, the Mann–Whitney U test identified significantly smaller bone volumes in patients who experienced complications. This finding suggests a possible threshold or nonlinear relationship. Notably, complication rates were higher in staged procedures than in simultaneous procedures, possibly reflecting lower preoperative bone volume in the former.

Our multivariable analysis shows that preoperative bone volume may be an independent predictor of atypical late complications following MSFA using bovine xenohybrid bone grafts. Even after adjusting for confounders such as graft volume, patient demographics, and procedural timing, an association persisted between lower bone volume and increased complication risk. This suggests that the native anatomical substrate plays a more critical role in long-term graft stability than the quantity of graft material itself.

Interestingly, although patients with complications received higher graft volumes on average, this variable lost significance in the multivariable model, implying a confounding relationship likely tied to the need for larger grafts in patients with greater bone deficiencies.

In addition, differences in occlusal force load between individuals—potentially influenced by factors such as gender, tooth position, and parafunctional habits—may also affect bone remodeling and graft integration. These biomechanical factors could contribute to the development of late complications and should be considered in future research. While patients with complications often received larger graft volumes, graft size itself did not independently predict adverse outcomes after adjusting for baseline bone conditions. These findings underscore that native bone availability—not graft quantity—is the more decisive factor for long-term MSFA success.

Our findings are partially consistent with those of previous studies [24,25,26]. While previous studies have primarily focused on peri-implantitis-related complications, we observed late complications unrelated to peri-implantitis. These findings align with those of a recent study describing late material-related complications [20]. The timeframe for complications in our cohort aligns with the previously reported late-onset failures after MSFA.

In a recent study, Ben-Dor et al. reported a 32% complication rate associated with the same grafting material used in sinus augmentation, with most cases requiring graft removal. Based on these findings, the authors concluded that the material is not recommended for sinus augmentation. While our study focuses on a different clinical context, their results highlight the importance of thorough preclinical and clinical evaluation. This topic remains highly relevant, and our findings further support the need for continued research into the appropriate indications and limitations of bovine xenohybrid bone grafts [16].

A noteworthy finding was the occurrence of atypical late complications that manifested several years after MSFA. Radiological data analysis indicated that these complications were not directly associated with peri-implantitis but may have been caused by material instability or delayed remodeling processes within the sinus floor.

The bovine xenohybrid bone graft (SmartBone^®^) used in this study is composed of a bovine bone-derived mineral matrix, reinforced with a PLCL copolymer coating and supplemented with RGD-exposing collagen fragments derived from animal gelatin. Due to local pH changes in the inflammatory phase of osseointegration, structural and chemical changes may take place in the polymer and/or the functional groups, potentially affecting the long-term stability of the material. Although several studies have examined the clinical and histopathological safety of PLCL and PLLA, none have reported preneoplastic or neoplastic reactions at the implantation site in humans. Pistner et al. previously described a long-term “intense resorptive histiocytic reaction” in humans implanted with crystalline block polylactide over 2 to 3 years of follow-up [25]. To mitigate such complications, recent advancements have incorporated intrinsically disordered proteins (smart bone peptides, SBP) in which NuPep peptide is embedded within the polymer coating of SBN. SBP shares the same composition as SB but includes IDPs (commercially known as NuPep, Corticalis AS, Oslo, Norway), which are physically entrapped within the polymeric coating during the manufacturing process, without undergoing any modifications or alterations. These intrinsically disordered proteins (IDPs) are resistant to processing with organic solvents and promote bone cell proliferation and differentiation. Furthermore, IDPs play a crucial role in biomineralization, particularly by regulating and directing mineral crystal growth [26]. Long-term clinical studies are required to further validate these findings.

This study is strengthened by detailed radiological evaluation using volumetric CBCT analysis, a clearly defined timeframe for complications, and rigorous statistical assessment. However, the study also had some limitations, including its retrospective design, a relatively small sample size (*n* = 47), and the inability to control for all confounding factors. Moreover, although histological analysis was performed, it was not reported systematically and was, therefore, excluded from this discussion to maintain consistency.

This study has several limitations. Its retrospective design makes it prone to selection bias, information bias, and missing data, limiting the ability to establish causality between preoperative bone volume and the occurrence of late complications. The small sample size (n = 47; 9 complications) may have been underpowered to detect statistically significant associations in multivariable analysis, increasing the risk of type II error. Although a trend toward more complications in patients with low preoperative bone volume was observed, this association did not reach statistical significance (*p* = 0.252) and should therefore be interpreted with caution. In addition, multiple implant brands, diameters, and placement protocols (simultaneous vs. staged) were used, which may introduce confounding effects that are difficult to fully control. The absence of a control group using alternative graft materials (e.g., autografts, allografts, or synthetic substitutes) further limits generalizability. Finally, follow-up durations varied among patients despite a median follow-up of 754 days, which may have led to underestimation of the true incidence of late complications.

In conclusion, this retrospective cohort study suggests that reduced preoperative bone volume may be associated with an increased risk of atypical late complications following MSFA using bovine xenohybrid grafts, although this association did not reach statistical significance. Our findings emphasize the importance of evaluating native bone volume before MSFA and implementing extended follow-up protocols beyond 2 years. Future prospective multicenter studies are necessary to validate these associations and reassess the long-term suitability of bovine xenohybrid bone grafts in anatomically compromised maxillary sinuses.

## Figures and Tables

**Table 1:** Univariate analyses of covariates vs. preoperative posterior bone volume (ROI).

Covariate	Test	Result	*p*-Value
Technical variables			
Graft amount (g)	Mann–Whitney U	1.0 (0.8–1.5) vs. 1.0 (1.0–1.0) mm^3^	0.5
Implants Length-to-Diameter Ratio	Mann–Whitney U	2.7 (2.5–2.8) vs. 2.7 (2.5–2.7)	0.9
Procedural variables			
Region (Regio)	Kruskal–Wallis	–	0.4
Implant timing (simultaneous vs. staged)	Mann–Whitney U	34.5 vs. 38.7 mm^3^	0.4
Clinical variables			
Pain	Mann–Whitney U	24.1 vs. 27.7 mm^3^	0.9
Fistula	Mann–Whitney U	24.6 vs. 5.2 mm^3^	0.3

Bone volume dichotomized at the median; continuous covariates compared by Mann–Whitney U [median (IQR) vs. median (IQR)]; categorical by Kruskal–Wallis or Mann–Whitney U; *p*-values > 0.1 rounded to one decimal place.

**Table 2:** Univariable Cox regression of covariates vs. time to complication.

Covariate	HR	95% CI	*p*-Value
Graft amount (per g)	1.49	0.46–4.86	0.5
Implants Length-to-Diameter Ratio	1.04	0.16–6.72	1.0
Implant timing (simultaneous vs. staged)	0.26	0.08–0.83	0.023
Pain	0.25	0.01–4.86	0.4
Fistula	1.00	0.00–491.80	1.0

Reporting HR [95% CI] and *p*-value; *p*-values > 0.1 rounded to one decimal place.

**Table 3:** Univariable Cox regression of preoperative bone volume (ROI) vs. atypical complications.

Covariate	HR	95% CI	*p*-Value
Preoperative bone volume (ROI)	0.99	0.96–1.01	0.3

Primary analysis of interest; reporting HR [95% CI] and *p*-value; *p*-values > 0.1 rounded to one decimal place.

**Table 4:** Multivariable Cox regression: predictors of atypical late complications using the exact interval from surgery to complication.

Covariate	Hazard Ratio (HR)	95% Confidence Interval	*p*-Value
Preoperative bone volume (ROI) (per 100 mm^3^ decrease)	0.97	0.93–1.02	0.25
Graft quantity (per 100 mm^3^)	1.02	0.95–1.09	0.46
Implant timing (staged vs. simultaneous)	0.91	0.39–2.15	0.83
Age (per year)	1.01	0.96–1.06	0.70
Sex (female vs. male)	0.50	0.18–1.44	0.20

For a supplementary sensitivity test, we also tested a flat-rate 12/36-month approach. Here, too, the same trend was confirmed (Preoperative bone volume: HR 0.979; 95% CI 0.934–1.025; *p* = 0.357; *p* = Gender (female vs. male): HR 0.781; 95% CI 0.300–2.030, *p* = 0.612).

## Data Availability

The original contributions presented in this study are included in the article. Further inquiries can be directed to the corresponding author.
